# Supplementary material for: Fattening Iberian Pigs Indoors vs. Outdoors: Production Performance and Market Value
Source: Animals (Basel). 2023 Jan 31;13(3):506. doi: 10.3390/ani13030506 (PMC9913101; doi:10.3390/ani13030506)
Supplement: Supplementary file 1 [file animals-13-00506-s001.zip › animals-2132011-supplementary.pdf]

animals

MDPI

Supplementary Materials:

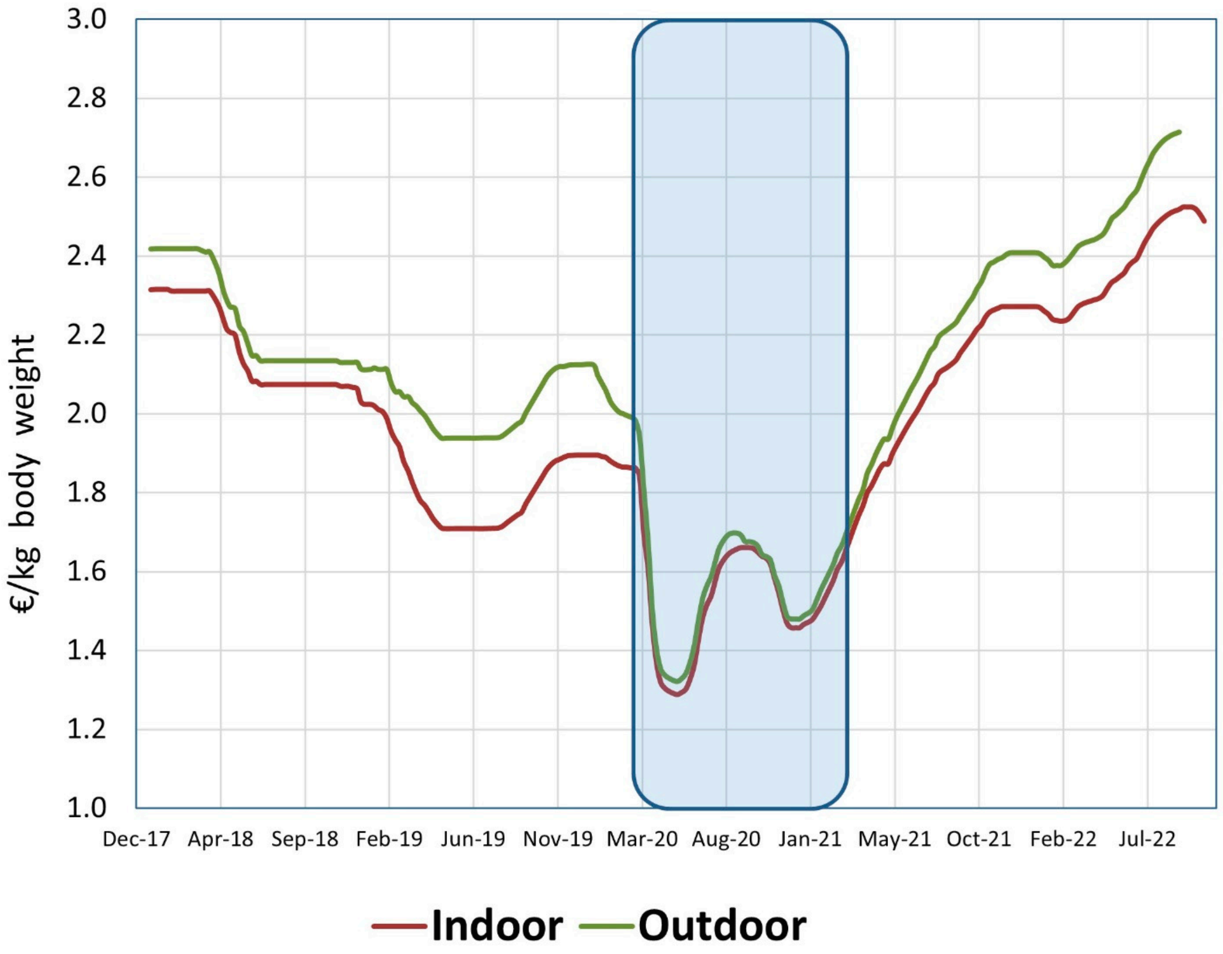

Figure S1: Evolution of the market price (€/kg body weight) of Iberian pigs in indoors and outdoors systems. Data from Lonja de Extremadura. The box represents the months considered as COVID.

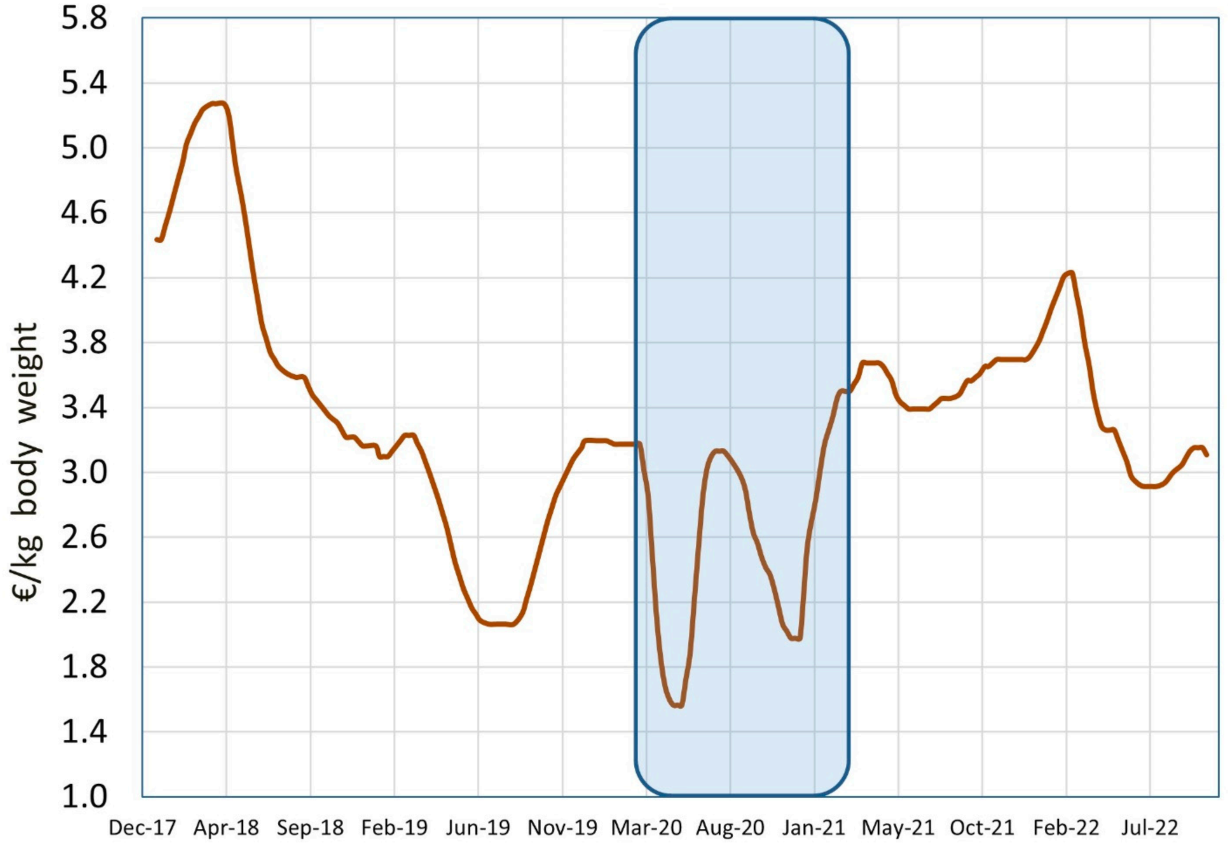

Figure S2: Evolution of the market price (€/kg body weight) of the Iberian piglet. Data from Lonja de Extremadura. The box represents the months considered as COVID

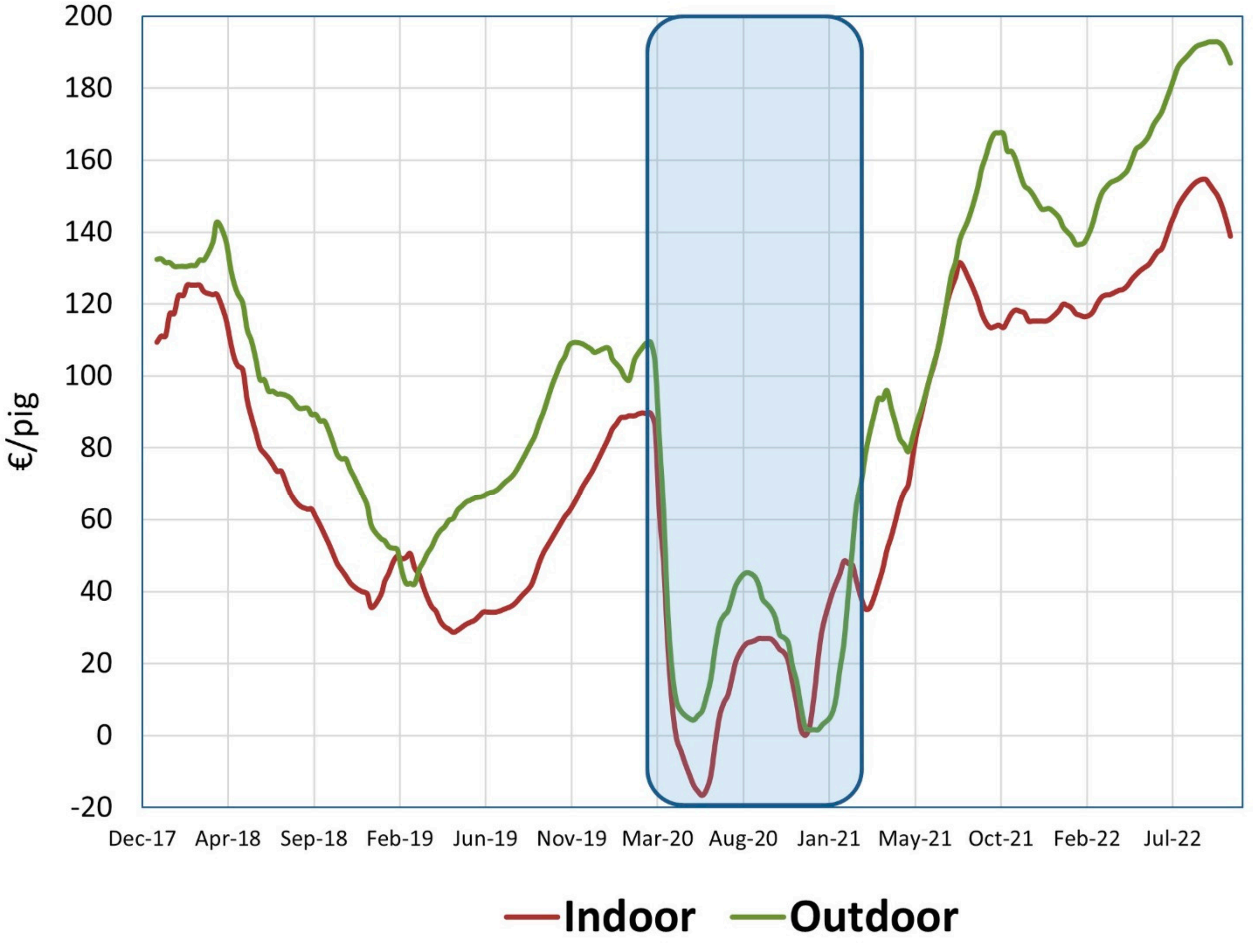

Figure S3: Evolution of gross profit (€/pig) of the Iberian pig in indoor and outdoor systems. Profits are calculated using expenditure data such as the price of the piglet (data from Lonja de Extremadura) and the cost of the feed consumed (data from our results) and the income is the value of the pig at the time of sale (data from Lonja de Extremadura). The box represents the months considered as COVID
